# App-based Self-administrable Clinical Tests of Physical Function: Development and Usability Study

**DOI:** 10.2196/16507

**Published:** 2020-04-27

**Authors:** Ronny Bergquist, Beatrix Vereijken, Sabato Mellone, Mattia Corzani, Jorunn L Helbostad, Kristin Taraldsen

**Affiliations:** 1 Department of Neuromedicine and Movement Science Norwegian University of Science and Technology Trondheim Norway; 2 Department of Electrical, Electronic and Information Engineering "Guglielmo Marconi" (DEI) University of Bologna Bologna Italy

**Keywords:** physical function, mHealth app, usability, older people, seniors

## Abstract

**Background:**

Objective measures of physical function in older adults are widely used to predict health outcomes such as disability, institutionalization, and mortality. App-based clinical tests allow users to assess their own physical function and have objective tracking of changes over time by use of their smartphones. Such tests can potentially guide interventions remotely and provide more detailed prognostic information about the participant’s physical performance for the users, therapists, and other health care personnel. We developed 3 smartphone apps with instrumented versions of the Timed Up and Go (Self-TUG), tandem stance (Self-Tandem), and Five Times Sit-to-Stand (Self-STS) tests.

**Objective:**

This study aimed to test the usability of 3 smartphone app–based self-tests of physical function using an iterative design.

**Methods:**

The apps were tested in 3 iterations: the first (n=189) and second (n=134) in a lab setting and the third (n=20) in a separate home-based study. Participants were healthy adults between 60 and 80 years of age. Assessors observed while participants self-administered the tests without any guidance. Errors were recorded, and usability problems were defined. Problems were addressed in each subsequent iteration. Perceived usability in the home-based setting was assessed by use of the System Usability Scale, the User Experience Questionnaire, and semi-structured interviews.

**Results:**

In the first iteration, 7 usability problems were identified; 42 (42/189, 22.0%) and 127 (127/189, 67.2%) participants were able to correctly perform the Self-TUG and Self-Tandem, respectively. In the second iteration, errors caused by the problems identified in the first iteration were drastically reduced, and 108 (108/134, 83.1%) and 106 (106/134, 79.1%) of the participants correctly performed the Self-TUG and Self-Tandem, respectively. The first version of the Self-STS was also tested in this iteration, and 40 (40/134, 30.1%) of the participants performed it correctly. For the third usability test, the 7 usability problems initially identified were further improved. Testing the apps in a home setting gave rise to some new usability problems, and for Self-TUG and Self-STS, the rates of correctly performed trials were slightly reduced from the second version, while for Self-Tandem, the rate increased. The mean System Usability Scale score was 77.63 points (SD 16.1 points), and 80-95% of the participants reported the highest or second highest positive rating on all items in the User Experience Questionnaire.

**Conclusions:**

The study results suggest that the apps have the potential to be used to self-test physical function in seniors in a nonsupervised home-based setting. The participants reported a high degree of ease of use. Evaluating the usability in a home setting allowed us to identify new usability problems that could affect the validity of the tests. These usability problems are not easily found in the lab setting, indicating that, if possible, app usability should be evaluated in both settings. Before being made available to end users, the apps require further improvements and validation.

## Introduction

At the time of retirement, at the age of 60-70 years, many people experience a significant decline in physical activity levels [1], and balance, gait, and mobility typically start to decline at a higher rate than before [2,3]. Thus, detection of changes in physical function at an early stage could be crucial to improve or prevent future declines in physical function and to sustain physical function over time. Objective assessment of physical function in health care settings is resource-demanding and therefore limited to people with a pressing need to have their function assessed, such as individuals who have experienced falls or who have been diagnosed with a condition known to affect physical functioning. Because functional decline typically occurs slowly, it might not pose an issue for the individual until their ability to perform activities of daily life is affected. Consequently, it might not be obvious why younger or well-functioning seniors should have their physical function assessed until it has come to this stage.

Innovations in mobile health (mHealth) technology have paved the way for new possibilities in assessing physical function. Most smartphones are equipped with sensors such as accelerometers, gyroscopes, and magnetometers and have high computational power; therefore, smartphones can be considered an inertial measurement unit enabling an objective and reliable assessment of physical function [4]. Considering that seniors are the fastest growing group of smartphone users [5] and that, in 2017, 42% of adults aged 65 or older in the United States owned a smartphone [6], there is great potential for using smartphones as a tool for self-assessing physical function [7]. Furthermore, well-designed and evidence-based apps represent new opportunities in preventive strategies for the senior population as a valuable tool in helping to make changes in their lives that can prevent functional decline.

Three such smartphone apps for self-assessment of physical function were developed as part of the PreventIT (early risk detection and prevention in aging people by self-administered ICT-supported assessment and a behavioral change intervention, delivered by use of smartphones and smartwatches) project. PreventIT was a European Union Horizon 2020 Personalising Health and Care project. The apps allow users to self-administer instrumented versions of the Timed Up and Go (Self-TUG), tandem stance (Self-Tandem), and Five Times Sit-to-Stand (Self-STS) tests in order to measure mobility and dynamic balance, static balance, and leg strength, respectively.

When developing an mHealth app for self-assessment of physical function, the usability of the app must be carefully considered, as it has been shown to be a fundamental determinant for technology adoption among older adults [8]. Usability is defined in the official International Organization for Standardization (ISO) guidelines as “the extent to which a product can be used by specified users to achieve specified goals with effectiveness, efficiency and satisfaction in a specified context of use” [9]. Furthermore, when measuring aspects of one’s health, the accuracy of the results relies on correct administration of the test. Thus, any usability problem associated with using an app-based test should be identified and addressed before it is made available to end users. This is usually done through several iterations of testing with target user groups, ideally until no major usability problems exist with regards to using the apps and administering the test. Usability studies are most often carried out in a lab setting, which is convenient and offers a high degree of control, as opposed to field-based usability testing. However, field-based testing, which, in this context, would be a home setting, provides insight into how the system is used under more realistic situations. Depending on the system being tested and the phase of development, usability should ideally be tested in both lab and home settings.

The overall aim of this study was to evaluate whether people in our target group of seniors between the ages of 60 years and 80 years were able to reliably self-administer the tests on their own using the smartphone, apps, and instructions we provided without any interaction with the assessors. In this paper, we describe the 3 iterations of usability testing with target user groups that were needed to identify all major usability problems. Each iteration consisted of a development phase and subsequent testing phase. In the first 2 iterations, we performed the usability tests in a controlled lab setting, where the assessors had prepared the test setup and necessary materials for the participants beforehand. For the third testing phase, participants were in their own homes, where they needed to prepare the test setup themselves by following instructions presented within the apps. This study does not address the topic of algorithms for signals and data processing nor how to present specific information and feedback to the users based on the test results.

## Methods

### Design Overview

We developed 3 app-based self-tests of physical function within the European Union Horizon 2020 project PreventIT [10]. Technology development in PreventIT followed the ISO standard 9241-210 [9] on user-centered development of products, and an iterative design approach was used to develop and test the usability of the apps. Because our target group is community dwellers and not clinical patients, we did not follow the ISO norm for medical devices. The target group of the apps was community-dwelling people aged 60 years and older, able to walk independently, and without any cognitive, functional hearing, or visual impairments. The overall aim of the mobile-based, self-administrable functional tests is early identification of risk for age-related functional decline by extracting relevant digital biomarkers from the smartphone-embedded inertial sensors. The intended context of use for the apps is to guide preventive intervention strategies for the general population.

An initial version (version 1) of the Self-TUG and Self-Tandem apps was included for the first iteration. The apps were upgraded based on the results of this testing, and the Self-STS was added as a third self-test. All 3 apps were tested under similar conditions during the second iteration (apps version 2). After further upgrades, version 3 of the apps was tested in a summative usability evaluation with a new group of volunteers in a home setting.

### Participants

We included participants from two studies. First, we included participants from a multicenter, 3-armed, feasibility randomized controlled trial conducted within the PreventIT project. For the first and second iterations, we included participants from the main study if they had performed the self-administration of the apps during baseline (iteration 1) and follow-up (iteration 2). The inclusion criteria for the participants are described in detail in the protocol paper for the PreventIT trial [10]. In short, for iterations 1 and 2, we included 189 and 134 community-dwelling adults, respectively, aged between 61 and 70 years from Trondheim, Norway; Stuttgart, Germany; and Amsterdam, the Netherlands.

For iteration 3, we included 20 community-dwelling adults ranging in age from 60 years to 80 years (mean 68.7 years, SD 5.2 years) in Trondheim, Norway. Inclusion criteria were community-dwelling status, age between 60 years and 80 years, ability to walk 500 meters independently, Norwegian-speaking, ability to hear sound from a smartphone, and current user of a smartphone. Participants were excluded if they reported any severe cardiovascular, pulmonary, neurological, or mental diseases.

### Description and Development of the Apps – From Version 1 to Version 3

We developed the apps using Android Studio 3.1.2 (Google, Mountain View, CA). Versions 1 and 2 of the apps were installed on a Samsung Galaxy S3 (Samsung, Seoul, Korea), while version 3 was installed on a Samsung Galaxy S8 (Samsung, Seoul, Korea).

#### Self-Timed Up and Go, Self-Sit to Stand, and Self-Tandem Apps

We created separate apps for each of the clinical tests (TUG, Five Times STS, and tandem stance). The apps were developed to be used as standalone tests, so one or more tests could be skipped if participants felt unsafe or did not want to perform a test. The TUG is a measure of mobility, in which the participant is timed while rising up from a chair, walking 3 meters, turning around, walking back, and sitting down again. In the Five Times STS, the participant is supposed to stand up from a chair and sit down again repeatedly 5 times as fast as possible, while being timed. In the tandem stance, the participant is supposed to place one foot in front of the other, heel-to-toe, in a straight line for 15 seconds, if possible. The Self-TUG uses an algorithm to detect the different phases of the TUG and the transitions between them (ie, sit-to-stand, walking, turning, turn-to-sit) from the sensor signals. Further, it calculates features from these phases, such as duration, velocity, jerkiness, and signal range, as well as gait features including number of steps, step duration, and gait speed. For the Self-STS, the algorithms analyze the sensor signals and calculate several features from the whole task, transitions, and separate sit-to-stand and stand-to-sit phases of each repetition. Finally, for Self-Tandem, the algorithms analyze the sensor signals and calculate features such as signal frequency, ellipse area, velocity, sway path, jerkiness, signal range, and spectral entropy.

#### Version 1

A multidisciplinary team designed the apps with emphasis on ease of use for the target group, corresponding to the term “perceived satisfaction” in the ISO terminology [9]. This included displaying buttons and icons in relatively large sizes and using contrasting colors on a white background. In addition, to ease the demands on working memory, the app screens were designed with as few elements and text as possible.

**Figure 1:**
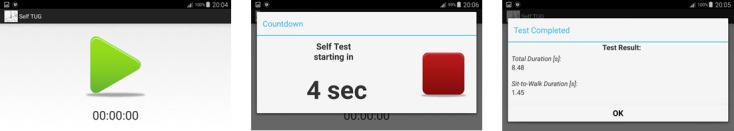
Screenshots of the first version of the Self-Timed Up and Go test.

All apps are based on the same structure (Figure 1). For example, when opening the self-TUG app, a green “play” button appears. Pressing the button prompts a dialog box with a 5-second countdown and a red stop button. The countdown gives the user time to attach the smartphone to the lower back by means of a waist belt case (see Procedures). After the countdown and as soon as no movement is detected by the inertial sensors, an audio signal initiates the start of the test. At the end of the test, when the user is again sitting still, an audio signal indicates that the test is completed. The Self-STS has the same structure (ie, audio signals for both the start and end of the test when the participant is sitting still). One important difference for the Self-Tandem is that the start and end signals are activated by time and not by movement. Thus, the audio start signal is initiated immediately after a 5-second countdown, followed by the end signal after 15 seconds.

#### Version 2

**Figure 2:**
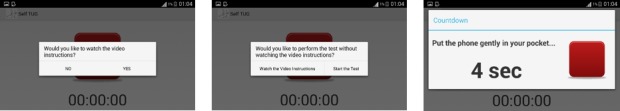
Screenshots of the second version of the Self-Timed Up and Go test.

In version 2 of the Self-TUG and Self-STS, the smartphone was worn in the front trouser pocket instead of the waist belt case. We also integrated instruction videos into the apps. By pressing “play,” a dialog box appears with a question asking whether the participants want to see the instruction video (with a yes/no choice; Figure 2). Pressing “yes” starts the instruction video for how to perform the self-test. Pressing “no” results in the question “Do you want to start the test?” with the options “Yes” or “No, play the instruction video.” A reminder of what to do (insert phone in pocket for Self-TUG and Self-STS, hold against chest for Self-Tandem) was added to the countdown dialog box. The apps were otherwise similar in structure as in version 1.

#### Version 3

The upgrades made for the third version of the apps included new instructional videos, with updated voiceover and footage, and new graphical elements in the video to emphasize important details of how to perform the tests (Multimedia Appendix 1) as well as a new menu structure where the user could choose to view instructions or start the test. Instructions consisted of a submenu with two options: watch the instructions for how to prepare the test setup or how to perform the test.

**Figure 3:**
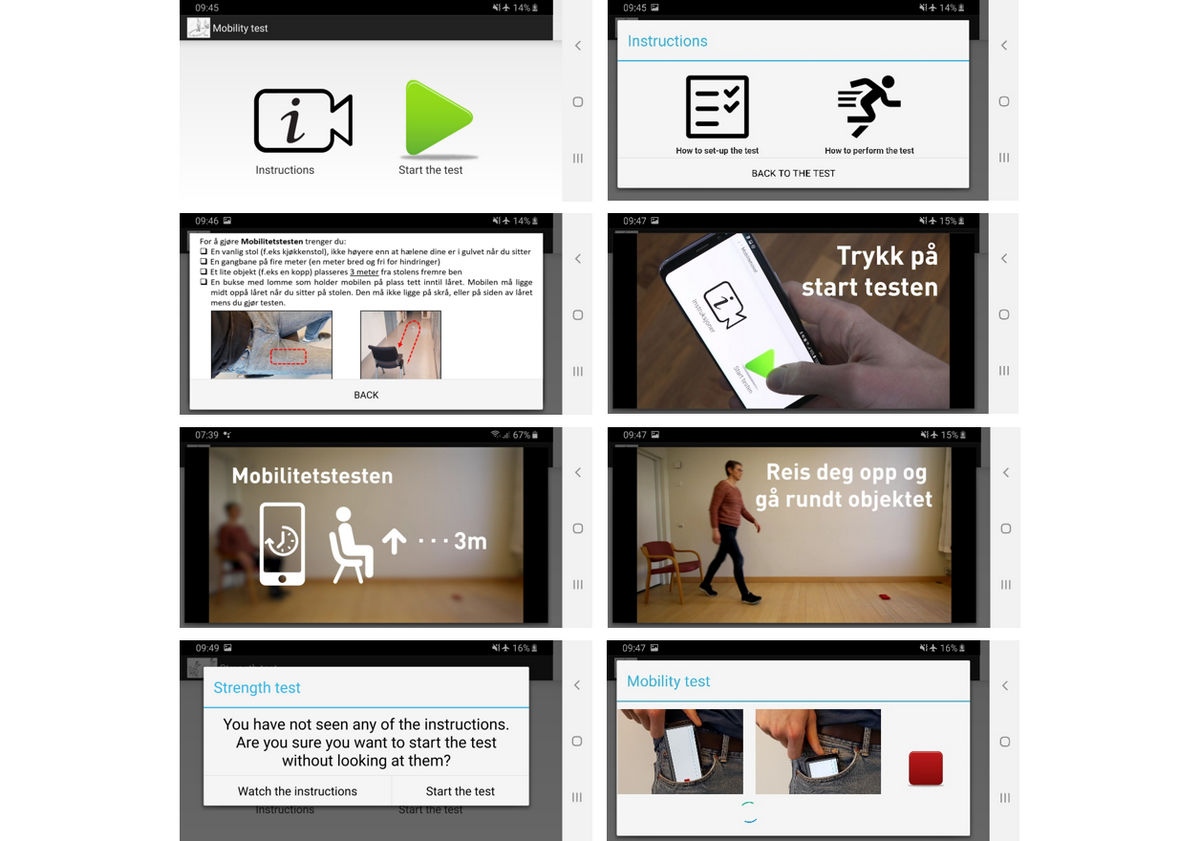
Screenshots of the third version of the Self-Timed Up and Go, including the start menu, instructions menu, test setup, instruction video screenshots, warning prompt when trying to start the test without having opened the instructions first, and instructions after starting the test.

In addition, new features (Figure 3) included a warning message that popped up if a user tried to perform a test without having watched both instructions; voiceover that instructed the user on what to do once the test sequence had been initiated (ie, “Put the phone gently in your right pocket. Sit down and wait for my instructions”); and real-time verbal feedback based on the inertial signals from the smartphone (eg, “sit down,” “get up from the chair,” “proceed with the test,” and count of repetitions for the Self-STS). The instruction videos were made for Norwegian study participants; thus, voiceover and text elements were in Norwegian. The text on the menu and dialog box was automatically adapted to the system language of the phone.

### Procedures

#### Testing of Version 1

The testing of version 1 was carried out in a lab setting by trained assessors. Before testing, the assessors prepared the setup, which included a chair placed against the wall with a 3-meter walkway in front of it and a beanbag at the 3-meter mark. Following a standardized introduction of the general purpose and procedures of a usability test, the participants were asked to self-administer the tests using the app on the smartphone. The participants did not receive any guidance from the assessors during the tests, and the materials they needed to perform the test were placed on a chair in front of them. This included the smartphone, written instructions, and a belt case for wearing the phone during the Self-TUG.

The assessors observed while the participants attempted to self-administer the tests, recording issues and errors on a sheet with predefined errors and issues that we expected in addition to a free-text box to record other errors and issues.

#### Testing of Version 2

For the second step, the Self-STS was added to the self-test battery. Testing happened under the same conditions as during testing of the first version, with one exception for the Self-TUG and Self-STS, namely that the smartphone placement was changed from the belt case to the front trousers pocket.

#### Testing of Version 3

An instructor visited the participants in their homes to get a realistic impression of how the system would be used in a real-world home setting. After a standardized introduction, the participants were asked to prepare and self-administer each of the 3 self-tests 3 times, without guidance from the instructor. One GoPro camera (GoPro, San Mateo, CA) was attached with a harness to the participant’s chest and worn during the test sequence to record the participant’s interaction with the smartphone. A second GoPro camera was placed in the room in a position where all movements could be recorded. The participants were encouraged to think aloud when using the system. After performing the self-tests, we asked participants to complete two questionnaires: User Experience Questionnaire (UEQ) and Systems Usability Scale (SUS) [11]. This was followed by an audiotaped semistructured interview that was developed specifically for this study, where we aimed to collect end users’ views on topics relevant to the apps, such as user experience, feedback/results, suggestions for improvements, and general usefulness of the apps.

### Data Processing and Analyses

We defined errors as deviations from the test instructions and counted the number of errors from the clinical record forms in the first and second iterations and from video recordings in the third iteration (Multimedia Appendix 2). SUS scores, based on 5-point Likert-scale items, were averaged for each participant and converted into a usability score with a range from 0 to 100 (with a higher score indicating better usability). UEQ Likert-scale items were scored from 0 (highly agree) to 4 (highly disagree), and frequencies of responses within each category across all items were calculated.

The interview transcripts were analyzed using thematic analysis [12] to identify relevant themes. Quotes were extracted for each subtheme and translated from Norwegian to English for analysis. The questions presented to the participants were “What did you think about using these apps to test your physical function?” and “Do you have any ideas for how the apps can be improved?”

### Ethics

The data collection was performed in accordance with the Helsinki ethical guidelines. The first and second usability testing phases were approved by the ethical committees in Norway (REK midt, 2016/1891), Stuttgart (registration number 770/2016BO1), and Amsterdam (METc VUmc registration number 2016.539 [NL59977.029.16]). The Norwegian Center for Research Data approved that the data protection for the third usability testing was in accordance with current regulations (ref. no. 391684). All participants included in this study gave their informed consent.

## Results

Participants’ characteristics are presented in Table 1.

**Table 1:** Participant characteristics.

Cohort	App version	n	Age (years), mean (SD)	Male gender, n (%)	Has smartphone experience, n (%)	Years of education, mean (SD)
PreventIT study	1	189	66.3 (2.4)	90 (47.4)	157 (83.1)	15.6 (4.6)
PreventIT study	2	134	66.3 (2.5)	64 (47.8)	108 (80.0)	15.9 (4.8)
Summative usability evaluation	3	20	68.7 (5.2)	11 (55.0)	20 (100.0)	—^a^

^a^Data were not collected.

The usability problems identified, numbers of participants who experienced these problems, and what was done to eliminate or reduce these problems are presented in Tables 2-4.

**Table 2:** Usability problems in version 1 of the Self-Timed Up and Go and Self-Tandem apps, rate of errors, and solutions (n=189).

Problem ID	Usability problem	Rate of errors/trials	Improvements made
1	Incorrect performance	120/378 (32%)	Added instruction video
2	Performed test without starting app	22/378 (6%)	Implemented instruction video that clearly demonstrates that the play button needs to be pressed before performing the test
3	Did not sit still and wait for start signal after test was started	23/189 (12%)	Added instruction video (demonstrating sitting still and waiting for the start signal before starting the test) and shortened the delay in the algorithm to limit any confusion
4	Incorrect placement of phone	32/378 (8%)	Changed placement to front pocket for Self-TUG^a^ and Self-STS^b^ and added a reminder in the countdown screen on what to do first (eg, “put the phone gently in your pocket”)
5	Did not hear/perceive instructions	18/378 (5%)	Changed placement to front pocket for Self-TUG and Self-STS
6	Accidentally cancelled the test	15/378 (4%)	Not possible to override the home button function in the android OS, change of placement the preferred solution to reduce this problem

^a^TUG: Timed Up and Go.

^b^STS: Sit to Stand.

**Table 3:** Usability problems in version 2 of all 3 self-tests, rate of errors, and solutions (n=134).

Problem ID	Usability problem	Rate of errors/trials	Improvements made
1	Incorrect performance	66/402 (16%)	Added new, improved instructions to the videos (new voiceover and added graphical elements to draw attention to the details of the test procedures); added a warning message that appears if trying to start the test without watching instructions; and added real-time TTS^a^ voice feedback on the number of repetitions in the Self-STS^b^
2	Started performing test (during instruction video) without starting the test in the app	28/402 (7%)	Changed structure of the app: main window now has two separate buttons, one for “start test” and one for “instructions”
3	Did not sit still and wait for start signal after test was started in the app	39/268 (15%)	Added real-time verbal step-by-step instructions that are initiated after the test is started in the app
4	Incorrect placement of phone	11/402 (3%)	Added real-time verbal instruction explaining where to place the phone and when to do this
5	Did not hear/perceive instructions	4/402 (1%)	Changed settings in the app so that the volume is always on maximum levels during testing, to prevent participants from accidentally pressing the “volume down” button
6	Accidentally cancelled the test	8/402 (2%)	Reduced the size of the “stop” button

^a^TTS: text-to-speech.

^b^STS: Sit to Stand.

**Table 4:** Usability problems identified in version 3 of all 3 self-tests and the rate of errors (n=20).

Problem ID	Usability problem	Rate of errors/trials
1	Incorrect performance	19/60 (32%)
2	Performed test (during instruction video) without starting the test in the app	5/60 (8%)
3	Did not sit still and wait for start signal after test was started in the app	0/40 (0%)
4	Incorrect placement of phone	0/60 (0%)
5	Did not hear/perceive instructions	2/60 (3%)
6	Accidentally cancelled the test	1/60 (2%)

### Iteration 1

In total, at least 1 error was made in 120 of 378 (32%) trials during the first usability testing with the Self-TUG and Self-Tandem. Forgetting or misunderstanding the written instructions were the leading causes of errors. In order to reduce the errors caused by this usability problem, we created video instructions to replace the written instructions.

### Iteration 2

In the second usability test, errors due to usability problem 1 (incorrect performance of test) were made in 66 of 402 trials (16%). Percentage of errors due to usability problems 2 (performs test without starting app) and 3 (did not sit still and wait for start signal after test was started) increased from the first usability test, while the frequency of problems 4-6 (incorrect placement of phone, did not hear/perceive instructions, accidentally cancelled the test, respectively) decreased.

### Iteration 3

In the third summative usability evaluation, errors due to usability problem 1 (incorrect performance of test) were made in 19 of 60 (32%) trials. Usability problems 3 (did not sit still and wait for start signal after test was started) and 4 (incorrect placement of phone) were eliminated, while the frequencies of usability problems 2 (performs test without starting app), 5 (did not hear/perceive instructions), and 6 (accidentally cancels the test) remained similar.

Table 5 presents an overview of the proportions of correctly performed (first) trials of self-tests for all tests in all iterations.

**Table 5:** Number of correctly performed self-tests (first trial) with versions 1, 2, and 3.

	Self-TUG^a^, n (%)	Self-STS^b^, n (%)	Self-Tandem, n (%)
Testing of version 1	42 (22.0)	N/A^c^	127 (67.2)
Testing of version 2	108 (83.1)	40 (30.1)	106 (79.1)
Testing of version 3	14 (70.0)	5 (25.0)	18 (90.0)

^a^TUG: Timed Up and Go.

^b^STS: Sit to Stand.

^c^Not yet developed.

### Perceived Ease of Use

UEQ scores for iteration 3 are presented in Table 6, indicating a positive or very positive user experience on all 6 items. Seven sub-themes of perceived ease of use were identified in the analysis of interview transcripts and are presented in Multimedia Appendix 3 with accompanying sample quotes, mapped to proposed solutions.

**Table 6:** Frequency of scores across the 6 items in the User Experience Questionnaire administered in the summative user evaluation (n=19).

Likert scale items	Strongly agree	Agree	Neither agree/disagree	Disagree	Strongly disagree
The set-up instructions were clear and easy to follow	14	4	1	0	0
The text in the app was easy to read	15	4	0	0	0
The buttons in the app were easy to discern from other elements	12	5	0	2	0
The signals were easy to hear	15	3	0	1	0
It was easy to navigate around in the apps	10	6	0	1	1
The instruction videos were clear and easy to follow	14	5	0	0	0

The mean score on the SUS for version 3 was 77.63 points (SD 16.1 points, range 42.5-97.4 points). Of the 20 participants, 14 participants scored the SUS above 66.5 points, which is the average SUS score for cell phones [13].

## Discussion

### Principal Findings

This paper describes the development and usability testing of the Self-TUG, Self-STS, and Self-Tandem through 2 iterations in the lab and 1 in a home setting. Our aim was to develop app-based, self-administrable tests of physical function that participants could use with a high degree of effectiveness and perceived ease of use. The first phase of testing revealed usability problems that affected the validity of the test results, illustrating a clear need for improvements. We addressed all usability problems by making changes to the app design, test algorithms, and test setup, which led to a large decrease in the number of trial errors in the second usability testing. The work on the third version of the apps then started, which included updating and adding new instructions for a version fully adapted for use in a home-based setting.

The results from the SUS, UEQ, and thematic analysis from the usability testing in the home setting indicated that the participants experienced high levels of perceived ease of use when using the apps. Still, errors were made that may affect the validity of the tests, most of which were caused by misunderstanding the instructions. As an example, the most common error for Self-STS was not performing it as fast as possible, which was the main reason why only 25.0% (5/20) performed it correctly on their first attempt. This is lower than in the second version tested in the lab (40/134, 30.1%). This misunderstanding was caused by a delay in the real-time counting of repetitions that was implemented in the third version of Self-STS. The verbal announcement of repetitions, which is also done by the assessor when the original Five Times STS is administered in the clinic, was implemented to motivate the participant to perform it faster and as a way for the participant to keep track. However, as can be seen in the sample quotes from the participant interviews (Multimedia Appendix 3), there was a delay in the real-time feedback, making many of the participants stop and wait in a standing position for the TTS to announce the repetition before sitting down. This slowed down the performance and thus impaired instead of improving the validity of the test.

During the Self-TUG, a common error was to measure an incorrect distance for the walkway during the set-up. Although the instructions state that the walkway should be 3 meters, the participant responses indicate that they did not consider it crucial to measure exactly 3 meters. However, it has to be exactly 3 meters if the total test duration, walking duration, or gait speed is to be used as an outcome measure, as these features rely on a standardized distance walked. A clarification in the instruction, where it is specified that the walkway needs to follow a straight line of exactly 3 meters, could be one way to increase the reliability of the test. However, as the distance walked by the participant cannot be accurately measured by the app, another approach could be to only exploit the distance-independent signal features, such as those calculated from the sit-to-stand, turning, and turn-to-sit phases. This will improve the system reliability in assessing motor performance, but it will not ensure full compatibility with the standard clinical measure of the total test duration.

Another common error with the Self-TUG was to press “Start test” without watching the instructions first. Although we had implemented a pop-up warning if this happened, a bug prevented this from happening in 5 of the 7 times this occurred. For the 2 participants who received the pop-up warning, 1 ended up watching the instruction video and performing the test correctly, while the other ignored the warning and proceeded to perform the test without watching the instruction video, thus performing it incorrectly. Because of the bug, we do not have sufficient information to make a safe claim regarding the effectiveness of the warning message. However, we assume that this problem will be resolved by fixing the bug and specifying in the warning message that a correct trial depends on having watched the instruction video first.

A common usability problem observed with the Self-Tandem, and also mentioned by many of the participants in the interviews, was the discrepancy between the instructions and actual duration required to stand in the tandem position. The instructions state that the participant is supposed to place the feet in tandem, hold the phone against the chest, and, after hearing the start signal, keep as still as possible for 15 seconds. What often happened in the current version, however, was that the app tried to detect and verify the position of the smartphone after the participant had been instructed to place the feet in a tandem position. The TTS then instructed the participant to hold the tandem position and keep as still as possible for 15 seconds, until the end signal. Depending on how fast they placed the phone against the chest, the participant could thus stand up to 25 seconds in total. However, if the instructions had said that the participant should assume the tandem position after hearing the start signal, different people would likely need a different amount of time to get into the correct position, thereby risking that we would get less than 15 seconds of actual tandem balancing. The outcome measure in Self-Tandem is mediolateral sway, which was found to be a strong predictor of age-related decline in a study in which an eyes-open condition was used [14]. We therefore designed the test in a way that would ensure, or at least increase the chance, that we would have at least 15 seconds of the participant standing in tandem.

A limitation with the Self-Tandem test is that we cannot infer whether the participant was keeping the correct tandem position for the entire 15 seconds from the inertial sensor signals. This is not true for the Self-STS and Self-TUG tests, where the correct performance of all phases of the test can be identified reliably from the signal. A potential solution could be to implement a pop-up question where the user self-reports whether they actually held the position for the entire duration. Such a solution has been implemented in the mHealth app “Steady” [14]. Steady is a falls risk app that consists of a health history questionnaire, 4 balance tasks (eyes open, eyes closed, tandem, and single leg), and a 30-second sit-to-stand test. The binary outcome measure of whether a user is able to complete a static steady-state balance task in various conditions and durations, such as those used in Steady, has been used extensively in studies assessing healthy young seniors [15].Therefore, adding this feature could potentially increase the Self-Tandem’s diagnostic/prognostic abilities.

### Implications and Future Work

The 3 iterations of usability testing described in this paper were sufficient to identify all major usability problems with the self-tests. The only problem remaining after the third cycle is the real-time counting in the Self-STS, described earlier in the discussion, which can easily be fixed.

We have demonstrated what challenges can be expected when developing app-based tests of physical function for seniors and how solutions to specific usability problems identified in one iteration affected the same problems in the next iterations (Tables 2-5). In addition, we described the perspectives of the seniors regarding their experience of using the apps to self-administer the tests (Multimedia Appendix 3). Another interesting insight is how going from the lab to a home-setting influenced the type of usability problems observed, in particular those related to the test setup. In the lab setting, the setup was prepared beforehand, whereas in the home setting, participants needed to follow the instructions in the app describing how to measure the walkway in the TUG, secure the chair for Self-STS, and perform the Self-Tandem in a spot with a secure object within hands reach, without any guidance from the assessor.

The next step in the developmental process of the apps is to implement the solutions proposed in Multimedia Appendix 3 to address the remaining usability problems and conduct new usability tests to ensure that the apps are ready to be used by the target group to self-administer the tests safely and correctly. Furthermore, the algorithms used for signal processing in Self-TUG and Self-STS need to be validated with the changed placement of the smartphone from the lower back (version 1) to the thigh (versions 2 and 3). Although we experienced some issues with this new placement in the usability testing (eg, some trousers were too loose), with the smartphone tilting down on either side of the thigh and making the trial invalid, we nevertheless believe that this solution offers the best trade-off between motion detection ability on one side and ease of use on the other side.

Our app-based tests of physical function could be applicable in many contexts, and different contexts may require different test outcomes. In the current version of the apps, the results presented to the user after performing the tests are total durations for the Self-TUG and Self-STS and sway path distance in the Self-Tandem. As discussed, however, these might not be feasible to exploit from an unsupervised test, where correct test set-up cannot be verified. The data processed by the inertial sensors within the smartphone provide additional features, and we aim to review existing literature to identify which of the signal features from instrumented versions of the TUG, Five Times STS, and standing tandem are the most predictive of functional decline in seniors. Given that these features can be reliably measured with the smartphone worn in the trouser pocket, they will be exploited as outcomes presented to the app users.

Although many tests of physical function have been instrumented by the use of smartphones, the authors are only aware of one other app that is developed for unsupervised self-assessment, the Steady app [14]. What separates the Steady app from ours is the type of tests implemented in the app. In addition to static balance and repeated sit-to-stands, we integrated the instrumented TUG. Furthermore, we performed usability assessments of the app in the participants’ own homes, in contrast to Steady, where an unoccupied apartment was used for all non-lab test sessions in order to mimic a home environment.

### Limitations

In our first 2 usability tests, the apps were tested by 189 and 134 participants, respectively. Although this was very useful for identifying what did and did not work well, we might have achieved similar results with fewer participants. Earlier studies have suggested that as few as 12 test users can be sufficient to detect the majority of usability problems [16]. Thus, with shorter and faster test cycles, the apps could potentially have been at a more mature stage today.

The participants in the summative usability evaluation differed to those from the PreventIT study in terms of age and smartphone experience. This makes it more difficult to say something about the impact that each app improvement had, as opposed to testing all app versions in 3 different, but homogenous, cohorts. However, we see it as an advantage that the apps are also tested in a slightly older cohort, as these participants can help us identify problems that could be more relevant to how they experience the usability of the apps, as compared to seniors that are younger or more experienced with technology in their daily life. Furthermore, the self-tests have not been validated in persons with tremor or pathologies; thus, the results do not necessarily generalize to these populations.

ISO’s definition of usability comprises 3 main aspects: effectiveness, which is the accuracy and completeness with which users achieve certain goals; efficiency, which is the relationship between the accuracy and completeness with which users achieve certain goals and the resources expended in achieving them; and satisfaction, which is the user’s comfort with and positive attitudes towards the use of the system [17]. Efficiency was not measured in our usability studies. It is often measured as task completion time or learning time, but in the context of testing physical function, where the time spent on completing a task also depends on the person’s physical abilities, we did not consider task completion time to be an appropriate outcome measure of usability, but rather of functional level of the participant.

Although we have assessed the usability of these apps and identified solutions to the remaining usability problems, the validity of the outcome measures from the tests also needs to be further investigated before being made available to end users. Another point worth mentioning is that the correct use of the apps and, accordingly, valid test results could be ensured by giving the end users a one-time demonstration of how to use apps and perform the tests correctly. This could be given in a home visit or in an appointment at the lab or clinic, depending on the context of use.

### Conclusion

The study results suggest that the apps have the potential to be offered as a solution for self-testing of physical function in a nonsupervised, home-based setting. Participants found the apps easy to use. The summative user evaluation in a home setting revealed important usability problems that were not identified in the lab, highlighting the importance of utilizing both test settings when assessing app usability. The current version of the apps has some remaining usability problems that can affect the test results, indicating that the apps need to be further improved and then validated before being made available to end users.

## Appendix

Multimedia Appendix 1 Video instruction available within version 3 of the Self-TUG app, demonstrating how to perform the Self-TUG, with voiceover in Norwegian.

Multimedia Appendix 2 Detailed description of usability problems.

Multimedia Appendix 3 Sub-themes within perceived ease of use and sample quotes from participant interviews following the third iteration of usability testing and proposed solutions.

## Abbreviations

ISO: International Organization for Standardization.
mHealth: mobile health.
STS: Sit-to-Stand.
SUS: Systems Usability Scale.
TUG: Timed Up and Go.
UEQ: User Experience Questionnaire.
