# Supplementary material for: App-based Self-administrable Clinical Tests of Physical Function: Development and Usability Study
Source: JMIR Mhealth Uhealth. 2020 Apr 27;8(4):e16507. doi: 10.2196/16507 (PMC7215517; doi:10.2196/16507)
Supplement: Multimedia Appendix 2 [file mhealth_v8i4e16507_app2.docx]

**Appendix 2**. Detailed description of usability problems

| **Problem ID** | **Problem category: performance of tasks** | **Description** |
| --- | --- | --- |
| 2 | Incorrect performance of task | Does not walk around object (TUG), does not sit down when returning to the chair (TUG), STS clearly not performed as fast as possible, not 5 reps, feet not fully extended |
| 6 | Starts performing test during instruction video | When seeing the phone being inserted into pocket in instruction video, participant start doing it themselves |
| 12 | Not able to keep tandem position for 15s | Moves feet and/or holds onto something during tandem |
| **Problem ID** | **Problem category: set-up** |  |
| 1 | Pocket not suitable for testing / real-time feedback not working | Pocket too short or too loose, preventing the phone to be kept in place on the thigh in TUG and STS. Leads to one or more of the following scenarios: 1) a seating position is not detected, which is required for the test sequence to start, and in order to detect that the user is finished (have sat down), 2) repetitions in STS not counted accurately, causing the participant to stop or slow down, and/or perform more than five repetitions. |
| 3 | Heels not in contact with floor in STS |  |
| 5 | TUG walkway incorrect | Walkway is curved, too long/short, or partly obstructed by furniture, or there's no object on the floor |
| 17 | No wall or chair for safety in tandem |  |
| 20 | Chair not secured in STS |  |
| **Problem ID** | **Problem category: app usability** |  |
| 7 | Did not perceive instruction | Did not hear clearly, e.g. due to speaker being squeezed, or impaired hearing |
| 8 | Presses the app icon more than once | Due to the delay of opening the app, the participant presses repeatedly, with the risk of pressing "instructions" or "start test" without knowing it |
| 9 | Struggles somewhat with opening the app | Pressing the button too long, moving the finger while pressing, pressing too lightly |
| 10 | Repeats instruction video instead of pressing "Back to test" | After having seen the instruction video the participant does not recognise how to start the test, and presses the "how to perform the test"-button again, as if he/she forgot that this is only the video, and that the test needs to be started in the previous menu. Category chosen when user clearly is looking for a way to stop the video, and not when participant watches video again on purpose. |
| 11 | Presses home button instead of back-button during video | participant presses home-button to stop the video, instead of the back-button |
| 13 | Accidentally presses "lock"-button while wearing phone in pocket |  |
| 14 | Not able to get back from results-screen without support |  |
| 15 | Presses "start test" without pressing "instructions" first | When opening app, participant ignores the "instruction"-button and directly presses the "start test"-button |
| 16 | Starts test without watching instructions first | Got warning, but started test anyway. "*" means warning did not show up (due to bug). |
| 18 | Accidentally cancels the test | E.g. pressing home-button |
| 19 | Other | Problems which can't be explained, bugs etc |
| 21 | Did not see the entire instruction video |  |
