# Supplementary material for: App-based Self-administrable Clinical Tests of Physical Function: Development and Usability Study
Source: JMIR Mhealth Uhealth. 2020 Apr 27;8(4):e16507. doi: 10.2196/16507 (PMC7215517; doi:10.2196/16507)
Supplement: Multimedia Appendix 3 [file mhealth_v8i4e16507_app3.docx]

**Appendix 3**. Sub-themes within perceived ease of use and sample quotes from participant interviews following the third iteration of usability testing and proposed solutions.

| **Sub-themes of perceived ease of use** | **Sample quotes** | **Solution** |
| --- | --- | --- |
|  |  |  |
| Apps were easy to use | *“I think it went surprisingly well.” ... “When you take the time to read the instructions, it’s okay” (M, 72)* |  |
| Unclear written instructions | *“The first app I think had too much text.” … “You have to read a lot before you understand what you are supposed to do”(M, 72)* | Video of set-up |
| Trying to start the test without reading and watching instructions first | *“I just, I think the start symbol was overwhelming. A green, large [arrow], so you became motived to push it right away” (M, 69)*  *“If you think of this as something you do regularly, I think it’s the first time you fumble with this, and then it will go very automatically” (M, 61)* | Insert text: “Step 1”, “Step 2”and “Step 3” above buttons for set-up, performance instructions and test, respectively. Also make the “Start test”-button grey until both instructions have been opened. |
| Real-time verbal counting of repetitions in Self-STS was unhelpful | *“It took a second or two before she said it [announced the repetition]. So it was she who controlled the tempo, in a way.” (M, 72)*  *“It seemed almost as if you had to slow down in order for the woman to keep up” (F, 63)*  *“I think it was unnecessary, actually. Because you are able to count to five.” (M, 63)*  *“If that was the goal [to do it as fast as possible], it turned out wrong” (M, 62)* | Enhance algorithm to detect and announce repetition sooner, or make the TTS announce only after all 5 repetitions have been performed. |
| Self-TUG walkway not accurately pre-measured | *“I thought that three meters, approximately three meters, that it was not a big case. If it is supposed to be exactly three meters, it should say so.” (M, 63)* | State in the set-up instructions that it is important that the walkway is exactly 3 meters. |
| Getting from instructions to test | *“...”go back”, then I think “have I done something wrong?”, then I have to go back to the beginning, in a way” (F, 72)*  *“To begin with I didn’t quite get it. Then, after familiarising myself with it, it wasn’t a problem” (F, 79)* | Place the “Start the test” button on the same menu as instruction buttons are. Make the button grey and unclickable, until both instructions have been opened; then it turns green and clickable. |
| Verbal instructions | *“I think it took a very, you were standing for a very long time in that [tandem] position before he said “now you are supposed to stand still”” (M, 65)*  *“I was instructed to place my feet in tandem, and by the time I was told that the test is starting, I had already been standing and keeping my balance” (M, 72)*  *“..because it took such a long time, that it actually, that I started to wonder whether I, if something was wrong, that I hadn’t pressed [the button] right” (M, 65)* | Enhance algorithms to reduce the waiting time. For the Self-Tandem: State in the instructions that the participant can use one hand to hold onto a chair or table until the TTS instructs the participant to let go. |
| App structure | *“So, you have three apps (sighs), will I scroll through them (?). If you have one app, which is easy to navigate within. There’s no reason for anything else, in my opinion” (M, 70)* | Integrate the three apps into a single app. |
